# Evaluation of EEG Oscillatory Patterns and Cognitive Process during Simple and Compound Limb Motor Imagery

**DOI:** 10.1371/journal.pone.0114853

**Published:** 2014-12-09

**Authors:** Weibo Yi, Shuang Qiu, Kun Wang, Hongzhi Qi, Lixin Zhang, Peng Zhou, Feng He, Dong Ming

**Affiliations:** 1 Department of Biomedical Engineering, Tianjin University, Tianjin, China; 2 Tianjin Key Laboratory of Biomedical Detecting Techniques and Instruments, Tianjin, China; University Medical Center Groningen UMCG, Netherlands

## Abstract

Motor imagery (MI), sharing similar neural representations to motor execution, is regarded as a window to investigate the cognitive motor processes. However, in comparison to simple limb motor imagery, significantly less work has been reported on brain oscillatory patterns induced by compound limb motor imagery which involves several parts of limbs. This study aims to investigate differences of the electroencephalogram (EEG) patterns as well as cognitive process between simple limb motor imagery and compound limb motor imagery. Ten subjects participated in the experiment involving three tasks of simple limb motor imagery (left hand, right hand, feet) and three tasks of compound limb motor imagery (both hands, left hand combined with right foot, right hand combined with left foot). Simultaneous imagination of different limbs contributes to the activation of larger cortical areas as well as two estimated sources located at corresponding motor areas within beta rhythm. Compared with simple limb motor imagery, compound limb motor imagery presents a network with more effective interactions overlying larger brain regions, additionally shows significantly larger causal flow over sensorimotor areas and larger causal density over both sensorimotor areas and neighboring regions. On the other hand, compound limb motor imagery also shows significantly larger 10–11 Hz alpha desynchronization at occipital areas and central theta synchronization. Furthermore, the phase-locking value (PLV) between central and occipital areas of left/right hand combined with contralateral foot imagery is significantly larger than that of simple limb motor imagery. All these findings imply that there exist apparent intrinsic distinctions of neural mechanism between simple and compound limb motor imagery, which presents a more complex effective connectivity network and may involve a more complex cognitive process during information processing.

## Introduction

Motor imagery (MI), defined as mental rehearsal of a motor act without any overt motor output, can modify the neuronal activity in the primary sensorimotor areas in a very similar way as motor execution [1]–[3]. In addition, motor imagery has also been demonstrated beneficial in motor rehabilitation in patients with movement disorders, and it plays a significantly important role in clinical and neuroscience studies [3]. Motor imagery can result in frequency specific changes of the ongoing EEG in forms of event-related desynchronization (ERD) or event-related synchronization (ERS), and the neural representations during mental tasks could be detected using spatial mapping of ERD/ERS. However, spatial mapping can not reveal the dynamic information flow between differently organized and specialized cortical regions [4]. Recently, there is a growing concern for interactions of the activated brain regions, typically in terms of “effective connectivity” [5]. Effective connectivity is a powerful method to analyze causal interaction among multiple neural regions in brain studies based on brain imaging techniques such as electroencephalogram (EEG).

As a result, the mutual interactions between different channels overlying core regions recruited by motor imagery could be revealed through effective connectivity network. However, most research has been concentrated to investigate the effective connectivity network induced by simple limb motor imagery involving a single limb. The effective connectivity networks over sensorimotor areas during hand or foot movement execution and imagination have been estimated by different methods such as Directed Transfer Function (DTF) and phase-locking value (PLV) [4], [6]–[8]. In contrast, significantly less work has been reported about brain oscillatory patterns induced by compound limb movement imagination [9], [10]. In addition, the effective connectivity network induced by compound limb motor imagery is still not clear. With respect to simple limb motor imagery, several parts of limbs like hand (forearm, postbrachium) and foot (shank, thigh) are involved in compound limb movement imagination, which may activate the neurons oscillation in multiple functional areas of cerebral cortex, and involve a different cognitive motor process. The question is that whether the effective connectivity network of compound limb motor imagery is more complex than that of simple limb motor imagery, at the same time, what are the differences of EEG oscillatory patterns and cognitive process between simple limb motor imagery and compound limb motor imagery.

In order to investigate the differences of the EEG patterns as well as cognitive process between simple limb motor imagery and compound limb motor imagery, seven kinds of mental tasks have been designed, involving three tasks of simple limb motor imagery (left hand, right hand, feet), three tasks of compound limb motor imagery combining hand with hand/foot (both hands, left hand combined with right foot, right hand combined with left foot) and rest state. Event-related spectral perturbation (ERSP), cortical source localization, effective connectivity network and phase synchronization analysis were adopted to analyze the time-frequency features, source locations, causal interactions, phase synchronization for different types of MI tasks.

## Materials and Methods

### Experimental procedure

**Figure 1:**
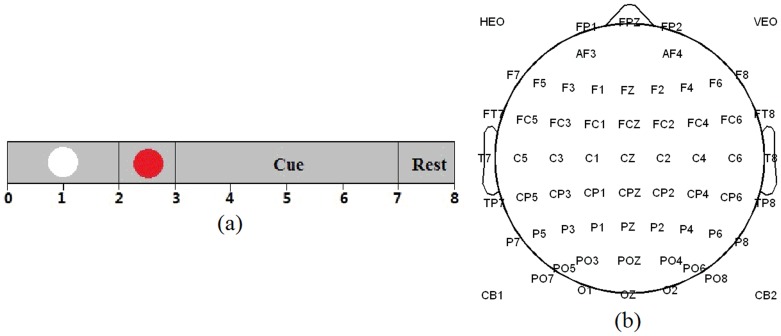
(a) Experimental paradigm of one trial. (b) 64-electrode positions.

Ten right-handed healthy subjects (7 females and 3 males, 23–25 years old) participated in this experiment [11]. All of the subjects had no prior experience with motor imagery before. Before EEG recording, they were required to train for one week to learn all MI tasks well. The subjects were sitting on a chair at one-meter distance from a computer screen. At the beginning of each trial (8 seconds), a white circle appeared at the center of the monitor. After 2 seconds, a red circle (preparation cue) appeared for 1 second to remind the subjects of paying attention to the character indication next. Then red circle disappeared and character indication (‘Left Hand’, ‘Left Hand & Right Foot’, et al) was presented on the screen for 4 seconds, during which the participants were asked to perform kinesthetic motor imagery rather than a visual type of imagery while avoiding any muscle movement. After 7 seconds, ‘Rest’ was presented for 1 second before next trial (Fig. 1(a)). The experiments were divided into 9 sections, involving 8 sections consisting of 60 trials each for six kinds of MI tasks (10 trials for each MI task in one section) and one section consisting of 80 trials for rest state. The sequence of six MI tasks was randomized. Intersection break was about 5 to 10 minutes.

EEG data was recorded from 64 Ag/AgCl scalp electrodes placed according to the International 10/20 System referenced to nose and grounded prefrontal lobe (Fig. 1(b)). The EEG signals were acquired by a Neuroscan SynAmps2 amplifier whose sampling rate is 1000 Hz and band-pass filtering range is 0.5–100 Hz. Besides, an additional 50-Hz notch filter was used during data acquisition. Thereafter, the original EEG signals were band-pass filtered between 0.5 and 50 Hz, and then downsampled at 200 Hz for following analysis.

The study was approved by the ethical committee of Tianjin University. All subjects signed informed consent in advance.

### Event-related spectral perturbation and source localization

The event-related spectral perturbation (ERSP) method allows us to inspect the spectral power changes of the induced EEG relative to the stimulus from the views of time-frequency domain, which could supply more details about ERD/ERS patterns of different types of motor imagery. Changes of event-related spectral power were analyzed with event-related spectral perturbation (ERSP) defined as follows:
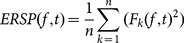
(1)where *n* is the number of trials, and 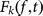 is the spectral estimation of *k*th trial at frequency *f* and time *t* [12]. ERSP values were calculated from −3000 ms to 5000 ms for every mental task. In this study, the averaged time-frequency ERD/ERS maps over all subjects from typical electrode positions were displayed between 1 and 40 Hz for analysis.

In addition, topographical distribution can help us figure out which areas are involved when ERD/ERS occurs during the imagination of different types of movements. Based on the ERSP values from 60 electrodes (except HEO, VEO, CB1 and CB2), the mean ERSP value in the specific frequency band and time interval was calculated for each electrode. Then, the single-subject mean ERSP values within beta band were subsequently averaged over all subjects to obtain group-level mean ERSP values, based on which the topography map for each MI task was obtained.

With the group-level mean ERSP values, the cortical source for each MI task could be estimated by solving the inverse problem from the scalp EEG signals to cortical source distribution. The cortical current density (CCD) source model was applied to solve the inverse problem using lead field WMN algorithm with the aid of the boundary element model [13]. The eConnectome software was used in this study to estimate the cortical source for each kind of motor imagery.

### Short-time directed transfer function

Directed transfer function (DTF), allowing for calculation of the causal interactions for arbitrary number of channels, was introduced by Kamiński and Blinowska [14]. The approach is based on a Multivariate Autoregressive model (MVAR) [15], through which the *k*-channel EEG signal 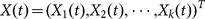 can be represented as
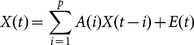
(2)where *A(i)* are the model coefficients, *E(t)* is a the vector of white noise values, *p* is the model order. Transforming the model coefficients into the frequency domain yields
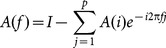
(3)


Directed Transfer Function (DTF) which describes causal influence of channel *j* on channel *i* at frequency *f* is defined as
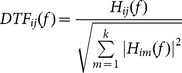
(4)where 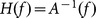. Equation (4) produces a ratio between the inflow from channel *j* to channel *i* and the joint inflows from all other channels to channel *i*, ranging from 0 to 1.

The Short-time DTF (SDTF) method relies on dividing the entire data epoch into short overlapping time intervals and to compute the DTF value for each interval [16]. In addition, zero-mean time series are required for MVAR model fitting. As a result, appropriate reprocessing is necessary to guarantee the stationary in short time intervals before the calculation of DTF [17]. The first step is to subtract the best-fitting line from each time series. The second step is to remove the temporal mean from each observation of the time series and divide the data by the temporal standard deviation over all channels. For multi-realization data, ensemble mean should be subtracted from every data point which also should be divided by ensemble standard deviation across trials. In this study, EEG signals, recorded from 21 electrodes that were chosen from 64 electrodes, were used to calculate the effective connectivity network. These 21 electrodes are overlying central and related brain areas, involving Fc3, Fc1, Fcz, Fc2, Fc4, C5, C3, C1, Cz, C2, C4, C6, Cp3, Cp1, Cpz, Cp2, Cp4, P3, P1, P2, P4. Considering the lack of bilateral coherence between both hemispheres [7], therefore the channels from different hemispheres could be evaluated separately and the following MVAR model was fitted simultaneously to 12 channels involving electrodes from right/left hemisphere plus midline electrodes Fcz, Cz, Cpz. The MVAR model coefficient was estimated by the method of Levinson, Wiggins, Robinson (LWR) algorithm [16], while the common optimal model order *p* was estimated by Bayesian Information Criterion (BIC). SDTF was evaluated for each MI task by sliding a short window in steps of 20% overlap, in which DTF was calculated for 100 samples long interval shifted by 20 samples. Therefore, we could obtain the time-frequency maps of SDTF for each combination of selected channels during simple and compound limb motor imagery. Statistical significance of the results was obtained by means of the bootstrap technique which can be used to evaluate the error of the estimated functions.

### Phase synchronization analysis

Phase synchronization is encountered in weakly interacting oscillator system and it manifests by the occurrence of a relationship between the corresponding phases variables [8]. At first, the original EEG signals were band-pass filtered within a specific frequency band. Then, the instantaneous phases of the signals could be extracted by Hilbert transformation, and the difference of instantaneous phases corresponding to two different signals was defined as 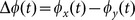. After obtaining the relative phases, the degree of synchronization between any two signals was evaluated, on a single trial basis, using the phase-locking value (PLV), defined as
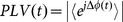
(5)where 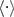 denotes the temporal average over a time interval. Thereafter, the mean PLV was computed by averaging over all trials for each kind of movement imagination. In this way, the PLV ranges from 0 (no synchronization) to 1 (phase synchronization), providing an indication on the degree of interaction between the two underlying systems.

In this study, phase synchronization was used to evaluate the underlying relationship between two brain regions, the central areas and the occipital areas, from which the signals were band-pass filtered between 4 and 5 Hz. Then, we averaged the signals from central areas including five electrodes C3, C1, Cz, C2, C4, while for the occipital areas, signals from Po7, Po5, Po3, Po4, Po6, Po8, O1, Oz, O2 were averaged. The PLV between two averaged signals was calculated in the time interval from 0 to 0.5 s. Permutation test was used at the 5% significance level.

## Results

### Event-related spectral perturbation

**Figure 2:**
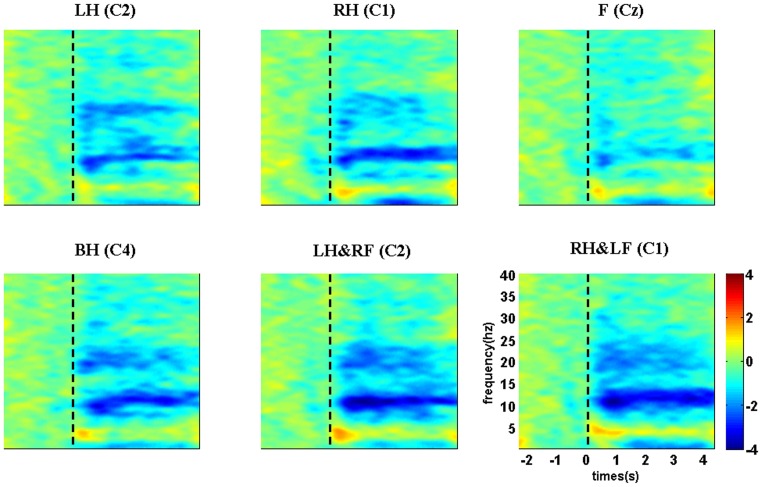
Examples of group-level average time-frequency maps at typical electrode locations for six MI tasks. LH, RH, F, BH, LH&RF, RH&LF indicate left hand, right hand, feet, both hands, left hand combined with right foot, right hand combined with left foot respectively. Blue indicates ERD and red indicates ERS, vertical dashed line indicates stimulus onset.

Fig. 2 shows the group-level average time-frequency maps at typical electrode locations for six MI tasks. The maps show obvious power decrease in both alpha and beta rhythms after stimulus onset. Moreover, taking a close look at the ERD patterns within alpha band, apart from the long-lasting ERD patterns located in 11–13 Hz, we also can see short-lasting ERD patterns located in 9–11 Hz within the first second especially for left/right hand imagery. Besides the induced alpha and beta ERD patterns, it also can be found that there exist theta (4–5 Hz) ERS patterns starting from stimulus onset lasting for about 0.5 seconds for all MI tasks. Details of short-lasting alpha ERD and theta ERS patterns are showed in section 3.4.

### Source localization analysis

**Figure 3:**
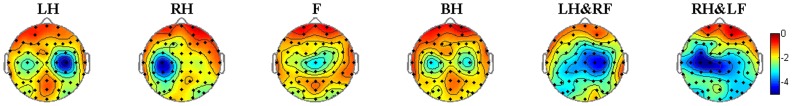
The topographical distribution of ERD patterns within beta band for six MI tasks. LH, RH, F, BH, LH&RF, RH&LF indicate left hand, right hand, feet, both hands, left hand combined with right foot, right hand combined with left foot respectively. Blue regions indicate the involved areas when ERD occurs during mental tasks.

Apart from the EEG signal analysis in time-frequency domain, spatial distribution analysis also plays an important role in exploring EEG patterns of different MI tasks. Here, we present the topographical distribution of ERD patterns within beta band for six MI tasks in Fig. 3. Different from the spatial distribution of single hand imagery, both left and right hand areas are desynchronized simultaneously during both hands imagery. From the distribution during left/right hand combined with contralateral foot imagery, we can see larger involved areas with strong ERD including contralateral hand area and midcentral area, which is obviously distinct from the distribution of simple limb motor imagery.

**Figure 4:**
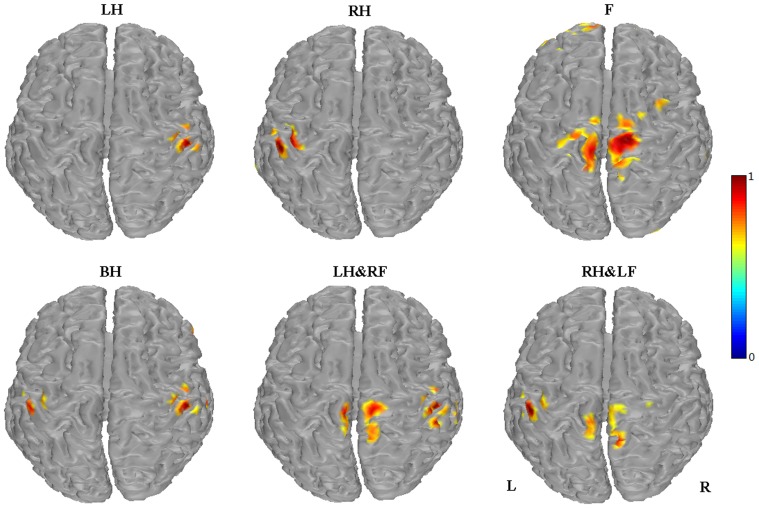
Source localization of induced beta ERD for six MI tasks. LH, RH, F, BH, LH&RF, RH&LF indicate left hand, right hand, feet, both hands, left hand combined with right foot, right hand combined with left foot respectively. Red regions indicate the estimated sources during mental tasks. L indicates left hemisphere and R indicates right hemisphere.

Based on the induced ERD values within beta band, the cortical source could be estimated to provide more accurate locations that are activated during different kinds of MI tasks. Fig. 4 shows the estimated sources of beta ERD induced by each MI task. As expected, visual discrimination between each other among these six MI tasks is apparent. The ERD sources are located in corresponding cortical areas somatotopically during simple limb motor imagery [18]–[20]. Compared with simple limb motor imagery, two estimated sources, located in bilateral hand areas, left hand area and foot area, right hand area and foot area, respectively, are observed during each imagination of compound limb movement, which indicates that compound limb motor imagery could activate corresponding representations of the imagined limbs.

### Effective connectivity network

**Figure 5:**
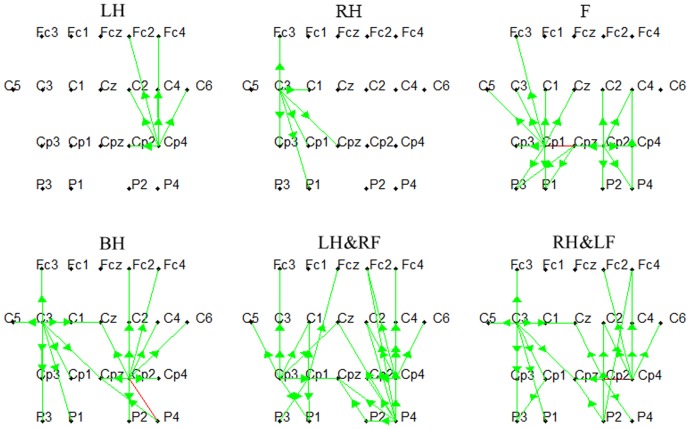
The networks of six different types of mental tasks. LH, RH, F, BH, LH&RF, RH&LF indicate left hand, right hand, feet, both hands, left hand combined with right foot, right hand combined with left foot respectively. Green line represents the unidirectional connectivity whereas the red line represents the bi-directional connectivity. The green arrows' tips point toward electrodes to which the flows are directed with their bases at ‘source electrodes’.

In order to reveal the dynamic information flow over activated brain regions, we integrated the SDTF value in the fixed frequency band and time interval during the performance of MI tasks within beta band. Fig. 5 shows the effective connectivity networks of six different types of MI tasks for one subject. In order to make the causal interaction between channels more clear, the amount of connectivity was limited by setting a threshold which was 60% of the maximum SDTF value. The outflows are showed by green arrows with their bases at ‘source electrodes’ and the tips pointing toward electrodes to which the flows are directed, whereas the red line represents the bi-directional connectivity. It can be found from Fig. 5, for the single hand imagery, the effective connections mainly exist in the contralateral hemisphere. The electrode Cp4 and C3 from which the outflows can be observed are the source electrodes respectively during left and right hand imagery. For the imagination of feet movement, the main outflows originate from Cp1, Cpz and Cp2 overlying the somatosensory areas. In addition, quite different from simple limb motor imagery, the characteristic feature of the network is the larger effective connectivity observed for compound limb motor imagery. More specifically, both hemispheres are involved and the outflows can be observed from both left and right brain regions (C3 and Cp2) during both hands imagery, which is apparently different from the flow patterns of single hand imagery. From the network of left/right hand combined with contralateral foot imagery, the outflows can be found not only from the central electrodes overlying sensorimotor areas (C3, Cp3, Cp1, Cp2, Cp4), but also from the electrodes from posterior parietal regions (P2, P4).

**Figure 6:**
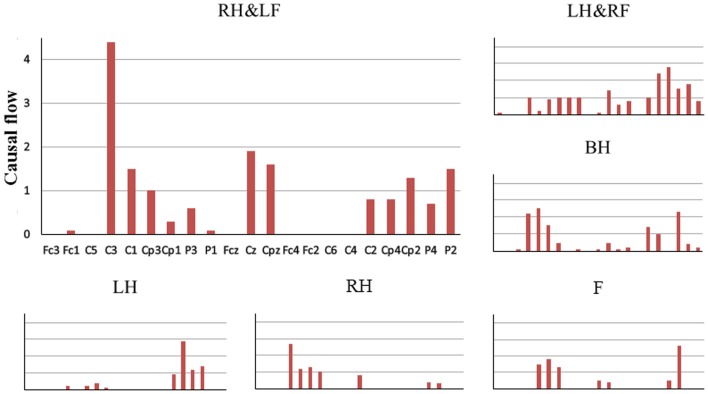
The mean positive causal flow of per electrode for six MI tasks. LH, RH, F, BH, LH&RF, RH&LF indicate left hand, right hand, feet, both hands, left hand combined with right foot, right hand combined with left foot respectively. Horizontal axis represents all 21 selected channels.

**Figure 7:**
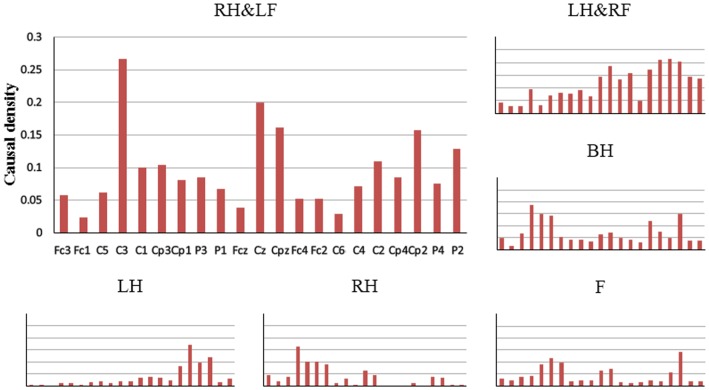
The mean causal density of per electrode for six MI tasks. LH, RH, F, BH, LH&RF, RH&LF indicate left hand, right hand, feet, both hands, left hand combined with right foot, right hand combined with left foot respectively. Horizontal axis represents all 21 selected channels.

Then, for quantitatively evaluating the networks of six MI tasks, causal flow, a parameter defined as the difference between out-degree (number of outgoing connections) and in-degree (number of incoming connections), was calculated for each node in the network. Fig. 6 shows mean positive causal flow of per electrode over all subjects for each MI task. Compared with left/right hand imagery, high causal flow can be observed from the electrodes overlying both left and right hand representations for both hands imagery. Meanwhile, different from simple limb motor imagery, more electrodes overlying sensorimotor areas with high causal flow can be found for left/right hand combined with contralateral foot imagery. We also calculated the causal density which measures the total amount of causal interactivity of a node. Mean causal density for each electrode over all subjects for each MI task is presented in Fig. 7. Similar phenomenon could be found for the electrodes over sensorimotor areas, additionally neighboring electrodes also show relatively higher causal density during compound limb motor imagery. Above phenomenon of causal flow and causal density indicate that more effective interactions are constructed in a larger network during compound limb motor imagery.

To further investigate the differences of the network between simple limb motor imagery and compound limb motor imagery, we constructed six groups for comparison of causal flow and causal density: BH vs LH, BH vs RH, LH&RF vs LH, RH&LF vs RH, LH&RF vs F, and RH&LF vs F. The paired *t*-test was used, and the *p* values that smaller than 0.05 in each column are in bold font. As showed in table 1, the electrodes with significant difference are overlying the sensorimotor areas, which means the causal flow of compound limb motor imagery is significantly higher than that of simple limb motor imagery at the specific electrode over sensorimotor areas. Table 2 shows the comparison of causal density for all selected channels. It can be observed that besides the electrodes over sensorimotor areas, compound limb motor imagery also performs significantly higher causal density at the electrodes over frontal areas and posterior parietal regions than simple limb motor imagery in each group, which indicates the differences of the network between simple and compound limb motor imagery.

**Table 1:** The electrodes with significant difference for the comparison of positive causal flow in six groups.

Causal flow	C3	C1	C4	C2	Cp2
BH vs LH	**0.045**	**0.046**	0.296	0.939	0.277
BH vs RH	0.748	0.093	**0.041**	0.053	**0.036**
LH&RF vs LH	0.172	0.500	0.466	0.678	0.363
RH&LF vs RH	**0.023**	0.348	0.172	0.111	**0.048**
LH&RF vs F	0.172	0.853	0.146	**0.023**	0.791
RH&LF vs F	**0.009**	0.500	0.105	0.111	0.857

The *p* values that smaller than 0.05 in each column are in bold font.

**Table 2:** The comparison of causal density for all selected channels in six groups.

Causal density	BH vs LH	BH vs RH	LH&RF vs LH	RH&LF vs RH	LH&RF vs F	RH&LF vs F
Fc3	**0.002**	0.339	**0.005**	0.097	0.097	**0.026**
Fc1	0.172	0.828	0.089	0.296	0.363	0.500
C5	**0.001**	**0.026**	**0.012**	0.089	0.828	0.069
C3	**0.017**	0.296	0.079	**0.007**	0.154	**0.003**
C1	**0.014**	0.136	0.069	0.500	0.845	0.445
Cp3	**0.005**	0.189	0.076	0.420	0.730	0.557
Cp1	**0.002**	0.948	0.076	0.591	0.704	0.627
P3	0.089	**0.012**	0.056	**0.035**	0.139	0.058
P1	**0.022**	0.248	**0.043**	**0.043**	0.100	**0.021**
Fcz	0.097	0.070	**0.007**	**0.012**	**0.009**	0.172
Cz	**0.021**	0.500	**0.001**	**0.009**	**0.016**	**0.014**
Cpz	0.068	0.070	**0.006**	**0.027**	**0.039**	0.127
Fc4	0.296	**0.001**	**0.030**	**0.009**	**0.008**	0.068
Fc2	0.321	**0.009**	**0.014**	**0.020**	**0.005**	0.054
C6	0.296	**0.012**	**0.026**	**0.041**	**0.005**	0.248
C4	0.187	**0.005**	0.097	**0.017**	**0.018**	0.074
C2	0.922	**0.005**	0.254	**0.013**	**0.003**	**0.033**
Cp4	0.941	0.379	0.076	0.110	**0.025**	0.257
Cp2	0.363	**0.045**	**0.022**	**0.004**	0.200	0.408
P4	0.122	**0.044**	**0.018**	**0.015**	**0.011**	**0.048**
P2	0.331	**0.033**	**0.018**	**0.006**	**0.005**	**0.009**

The *p* values that smaller than 0.05 in each column are in bold font.

### 10–11 Hz alpha ERD and theta ERS patterns

For the extra findings from time-frequency maps, we gained the topographical distribution based on the 10–11 Hz ERD patterns between 0.5 and 1 second for each MI task. As revealed in S1 Figure, besides from the ERD feature observed on the sensorimotor areas, the occipital areas are desynchronized as well for all MI tasks. With respect to the ERD over occipital areas, the ERD values from 9 electrodes including Po7, Po5, Po3, Po4, Po6, Po8, O1, Oz, O2 were averaged for comparison in six groups as above. S2 Figure indicates that compound limb motor imagery shows a significantly larger ERD at occipital areas as compared to simple limb motor imagery.

For the theta ERS, we also calculated spatial distribution within 4–5 Hz between 0 and 0.5 second for each MI task. As displayed in S3 Figure, two main brain regions, the central areas and the occipital areas, are synchronized for all MI tasks. However, the ERS found in the central areas during compound limb motor imagery seems stronger than that of simple limb motor imagery. The ERS values from five electrodes including C3, C1, Cz, C2, C4 were averaged for similar comparison. The statistical results presented in S4 Figure for both the Cz position and the central areas show that the theta ERS values induced by compound limb motor imagery are significantly larger than those of simple limb motor imagery.

Furthermore, the underlying relationship between central areas and occipital areas was evaluated by phase-locking value and compared between simple and compound limb motor imagery in the way as above. The statistical result presented in S5 Figure indicates that the PLV of right hand combined with contralateral foot imagery is significantly larger than that of right hand imagery and feet imagery, and additionally the PLV of left hand combined with contralateral foot imagery is significantly larger than that of feet imagery.

## Discussion

Simultaneous movement imagination of different limbs contributes to the result that corresponding motor areas are activated simultaneously. In addition, source localization provides more convincing proof that two different motor areas, corresponding to the limbs involved in different kinds of compound limb motor imagery, are activated as expected. Although the strongest beta ERD appears on the corresponding limb areas, larger brain regions are involved during left/right hand combined with contralateral foot imagery. Such phenomenon means more neurons are activated in frontoparietal regions due to the combination of hand and foot movement.

Source localization can only reveal the difference of activation pattern over sensorimotor areas between simple and compound limb motor imagery, while effective connectivity network can explore the dynamic mutual interaction between sensorimotor areas and neighboring regions. From the effective connectivity networks, the most important finding is the new pattern of EEG flow concerning the dynamics of the information processing during compound limb motor imagery, whose pattern of interactions among brain regions is totally different from that of simple limb motor imagery. Because of the simultaneous imagination of both hands, both hemispheres are activated and the effective connections appear on both left and right hand areas simultaneously while only contralateral hand areas is involved during single hand motor imagery. Larger regions overlying the sensorimotor areas are activated in contrast to simple limb motor imagery due to the involvement of upper limb and contralateral lower limbs together. Meanwhile, more causal sources are verified for compound limb motor imagery by statistical analysis of causal flow (a node with a highly positive flow is a causal ‘source’) [21]. Both causal flow analysis and source localization present a similar result that compound limb motor imagery activates more functional regions over sensorimotor areas. In addition, the other difference is the observed increasingly more effective interactions overlying larger brain regions besides sensorimotor areas for compound limb motor imagery, which is demonstrated by statistical analysis of causal density, a useful measure of dynamical complexity [21].

**Figure 8:**
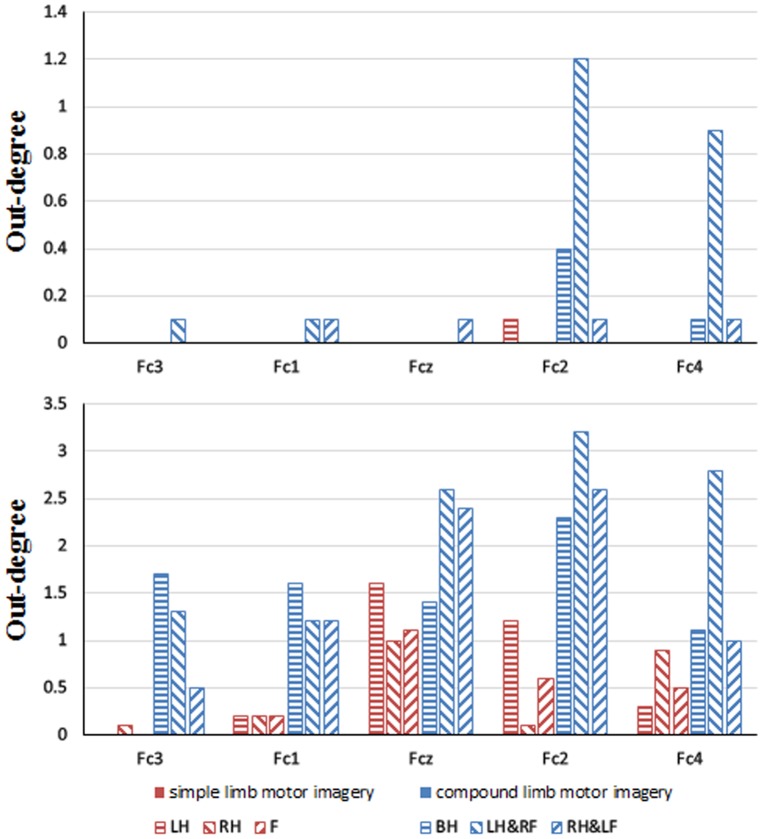
The mean out-degree of the midfrontal electrodes under a 60% threshold (up) and under a 30% threshold (bottom) for six MI tasks. Red bar indicates simple limb motor imagery, while blue bar indicates compound limb motor imagery. LH, RH, F, BH, LH&RF, RH&LF indicate left hand, right hand, feet, both hands, left hand combined with right foot, right hand combined with left foot respectively. Horizontal axis represents 5 selected channels.

The networks highlight the interactions over sensorimotor areas while no outflow is observed from midfrontal electrodes due to the setting of a 60% threshold. In order to further explore the EEG flow over frontal cortex, we calculated the out-degree for Fc3, Fc1, Fcz, Fc2 and Fc4 over all subjects under a 60% threshold as well as under a 30% threshold. As showed in Fig. 8, by decreasing the threshold, more outflows can be detected from midfrontal electrodes for each MI task under a 30% threshold. Thus it can be seen that the networks could present different flow patterns depending on setting different thresholds. In addition, compound limb motor imagery presents larger out-degree than simple limb motor imagery for the midfrontal electrodes under a 30% threshold by statistical analysis, which demonstrates the difference in the activation of frontal cortex between simple and compound limb motor imagery.

**Figure 9:**
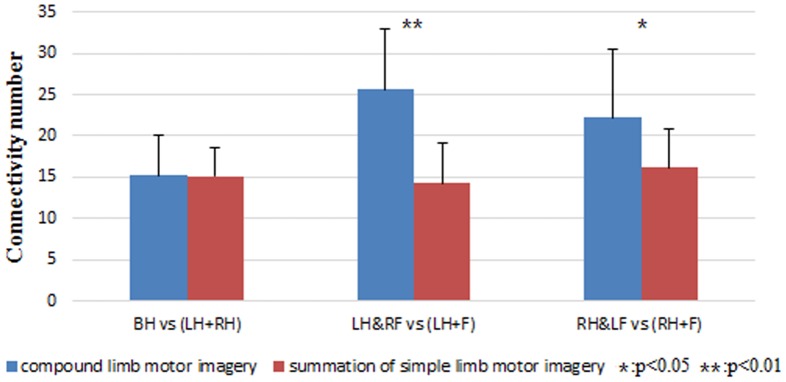
The comparison between connectivity number in the network of compound limb motor imagery and the summation of connectivity number in the networks of corresponding simple limb motor imagery. Blue bar indicates compound limb motor imagery, while red bar indicates the summation of simple limb motor imagery. (LH+RH) indicates the summation of left hand imagery and right hand imagery. (LH+F) indicates the summation of left hand imagery and feet imagery. (RH+F) indicates the summation of right hand imagery and feet imagery. Condition pairs that significantly differ from each other are indicated by an asterisk (*p*<0.05) or two asterisks (*p*<0.01).

From the result of causal flow analysis and source localization, the activation of sensorimotor areas during compound limb motor imagery seems to be the summation of that during two kinds of simple limb motor imagery. However, through calculating the number of all connectivity in the networks, it can be found in Fig. 9 that the connectivity number of left/right hand combined with contralateral foot imagery is significantly larger than the summation of connectivity number observed in left/right hand imagery and feet imagery respectively. Therefore, the patterns of EEG flow are probably not the simple linear superposition of information flow generated by different limbs during compound limb motor imagery. More interactions between the electrodes overlying sensorimotor areas with the addition of frontal cortex and posterior parietal cortex may be the result of the involvement of a larger neural network or more cell assemblies in information processing during compound limb motor imagery. In particular, frontal cortex is especially important for integrating complex perceptual information from sensory and motor cortices to perform cognitive tasks [22]. Whereas, the interactions within posterior parietal cortex are likely to reflect such higher cognitive functions as updating postural representations of the limbs [23]. As a result, compared with simple limb motor imagery, a more complex cognitive process may occur during compound limb motor imagery, whose effective connectivity network is more complex and involves a broader range of information exchanges between sensorimotor areas and neighboring regions.

As a therapeutic tool in neurological rehabilitation, motor imagery with the impaired limb could induce increased ipsilateral activation in primary motor area and supplementary motor area in stroke patients, which indicates that ipsilateral human motor structures might compensate functionally for contralateral motor cortex dysfunction. The essence of motor imagery in neural rehabilitation is to facilitate the reorganization of the affected areas and loops by recruiting intact neurons and strengthening activity in other neuronal loops [3]. Therefore, it can be inferred that the activation of larger cortical areas as well as the enhancement of information exchanges between different functional areas probably implies the feasibility and benefit for the application of compound limb motor imagery, which may combine impaired limb with intact limb in motor rehabilitation after neural injury.

On the other hand, we also investigated the difference of EEG oscillatory patterns based on the extra findings from time-frequency maps. As we know, upper alpha desynchronization (about 10–12 Hz) is very often topographically restricted and develops during the processing of sensory-semantic information above parieto-occipital areas [24]–[26]. Therefore, the 10–11 Hz ERD found at occipital regions may result from words understanding of the cues occurring in the experiment. Significantly larger ERD at occipital regions can be interpreted as the higher mental effort occupied in retrieval processes for semantic information corresponding to compound limb motor imagery since the degree of desynchronization is closely linked to semantic memory processes.

**Figure 10:**
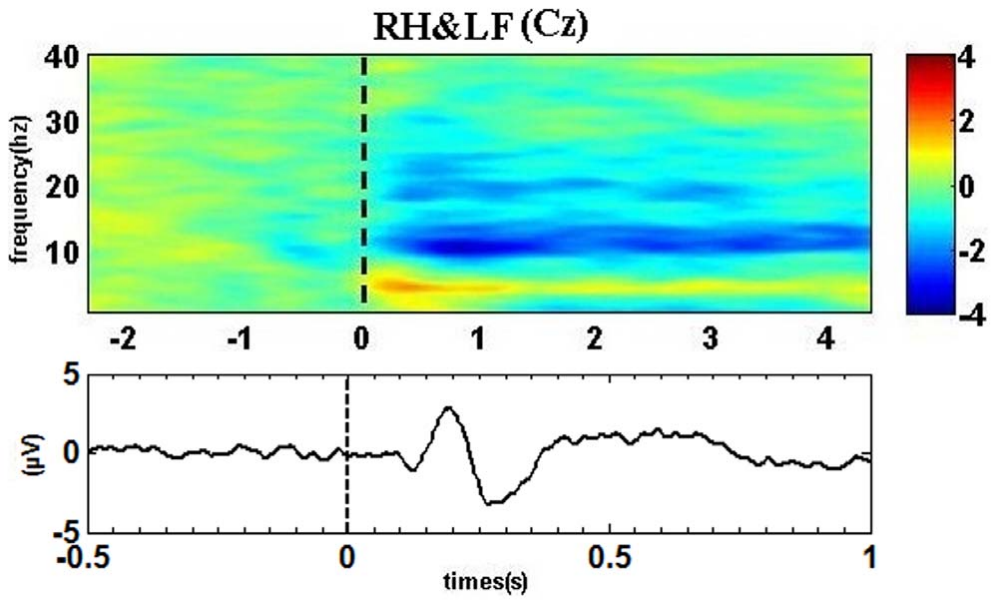
The example of group-level average time-frequency map (up) and VEP (bottom) at electrode Cz for right hand combined with left foot imagery. Blue indicates ERD and red indicates ERS. Vertical dashed line indicates stimulus onset.

The cue stimulus could induce the visual evoked potential (VEP) components. Fig. 10 shows the example of time-frequency map and VEP at electrode Cz for right hand combined with left foot imagery. A positive peak can be found at ∼200 ms. Statistical analysis for peak values in six comparison groups by *t*-test shows that significant difference (*p* = 0.02) can only be found between both hands imagery and right hand imagery. In contrast, compound limb motor imagery shows significantly larger central theta synchronization for all comparison groups (S4 Figure). Therefore, the VEP components may have influence on the generation of theta synchronization, but are not the main factors. An enhancement of theta occipital rhythm may owe to the successfully encoding of cues consisting of words, since the theta synchronization is positively correlated with the ability to encode new information [27]. At the same time, the central ERS is likely to reflect the establishment of a bottom up pathway to retrieve the movement-related experiences stored in the sensorimotor cortices while extracting the meaning of the perceived information and preparing the simulated movement [28], [29]. Therefore, significantly larger central ERS may indicate a higher level of attention and alertness is required during encoding the information corresponding to compound limb motor imagery. Furthermore, significantly lager PLV reveals a closer collaborative relationship between central and occipital areas for left/right hand combined with contralateral foot imagery, which probably implies more attentional demands during the process of the bottom up pathway activation while preparing a more complex movement. As a consequence, the differences found in 10–11 Hz alpha ERD and theta ERS patterns between simple and compound limb motor imagery probably also reveal the complexity of the cognitive process during compound limb motor imagery.

## Conclusion

This study investigated the differences of the EEG oscillatory patterns between simple limb motor imagery and compound limb motor imagery from multiple angles using different methods, revealed a more complex cognitive process for compound limb motor imagery. Cortical source localization showed activated brain regions corresponding to different limbs, while effective connectivity network revealed a more complex effective connectivity network for compound limb motor imagery. The patterns of EEG flow are probably not the simple summation of information flow generated by different limbs during compound limb motor imagery, whose effective connectivity network involves a larger range of information exchanges between sensorimotor areas and neighboring regions.

## Supporting Information

S1 Figure **The topographical distribution of 10–11Hz ERD for six MI tasks.** LH, RH, F, BH, LH&RF, RH&LF indicate left hand, right hand, feet, both hands, left hand combined with right foot, right hand combined with left foot respectively. Blue regions indicate the involved areas when ERD occurs during mental tasks.
(TIF)

S2 Figure **The comparison of ERD values in six groups for occipital areas.** Blue bar indicates compound limb motor imagery, while red bar indicates simple limb motor imagery. Condition pairs that significantly differ from each other are indicated by an asterisk (*p*<0.05) or two asterisks (*p*<0.01).
(TIF)

S3 Figure **The topographical distribution of ERS patterns within theta band for six MI tasks.** LH, RH, F, BH, LH&RF, RH&LF indicate left hand, right hand, feet, both hands, left hand combined with right foot, right hand combined with left foot respectively. Red regions indicate the involved areas when ERS occurs during mental tasks.
(TIF)

S4 Figure **The comparison of ERS values in six groups for Cz and central areas.** Blue bar indicates compound limb motor imagery, while red bar indicates simple limb motor imagery. Condition pairs that significantly differ from each other are indicated by an asterisk (*p*<0.05) or two asterisks (*p*<0.01).
(TIF)

S5 Figure **The comparison of the PLV between central and occipital areas in six groups.** Blue bar indicates compound limb motor imagery, while red bar indicates simple limb motor imagery. Condition pairs that significantly differ from each other are indicated by an asterisk (*p*<0.05) or two asterisks (*p*<0.01).
(TIF)

## References

[1] Pfurtscheller G, Neuper C (1993) Motor imagery and direct brain–computer communication. Proceeding of the IEEE 89:1123–1134.

[2] Pfurtscheller G, Neuper C (1997) Motor imagery activates primary sensorimotor area in humans. Neurosci Lett 239:65–68. 9469657 10.1016/s0304-3940(97)00889-6

[3] Munzert J, Lorey B, Zentgraf K (2009) Cognitive motor processes: the role of motor imagery in the study of motor representations. Brain Res Rev 60:306–326. 19167426 10.1016/j.brainresrev.2008.12.024

[4] Athanasiou A, Lithari C, Kalogianni K, Klados MA, Bamidis PD (2012) Source Detection and Functional Connectivity of the Sensorimotor Cortex during Actual and Imaginary limb movement: a preliminary study on the implementation of econnectome in motor imagery protocols. Advances in Human-Computer Interaction Volume doi:10.1155/2012/127627

[5] Friston KJ (1993) Time-dependent changes in effective connectivity measured with PE. Hum Brain Mapp 13:69–79.

[6] Ginter J Jr, Blinowska KJ, Kamiński M, Durka PJ, Pfurtscheller G, et al (2005) Propagation of EEG activity in beta and gamma band during movement imagery in human. Method Inform Med 44:106–113. 15778801

[7] Kus R, Ginter J Jr, Blinowska KJ (2006) Propagation of EEG activity during finger movement and its imagination. Acta Neurobiol Exp 66:195–206. 10.55782/ane-2006-1607 17133951

[8] Stavrinou ML, Moraru L, Cimponeriu L, Della-Penna S, Bezerianos A (2007) Evaluation of cortical connectivity during real and imagined rhythmic finger tapping. Brain Topogr 19:137–145. 17587169 10.1007/s10548-007-0020-7

[9] Zhou Z, Wan B, Ming D, Qi H (2010) A novel technique for phase synchrony measurement from the complex motor imaginary potential of combined body and limb action. J Neural Eng 7:046008. 20571185 10.1088/1741-2560/7/4/046008

[10] Doud J, Lucas P, Pisansky T, He B (2011) Continuous three-dimensional control of a virtual helicopter using a motor imagery based brain–computer interface. PloS one 6:e26322–26331. 22046274 10.1371/journal.pone.0026322 PMC3202533

[11] Yi W, Qiu S, Qi H, Zhang L, Wan B, et al (2013) EEG feature comparison and classification of simple and compound limb motor imagery. J Neuroeng Rehabil 10:106. 24119261 10.1186/1743-0003-10-106 PMC3853015

[12] Delorme A, Makeig S (2004) EEGLAB: an open source toolbox for analysis of single-trial EEG dynamics. J Neurosci Meth 134:9–21. 10.1016/j.jneumeth.2003.10.009 15102499

[13] He B, Astonlfi LBF, Han Y, Yang L (2011) eConnectome: A MATLAB toolbox for mapping and imaging of brain functional connectivity. J Neurosci Meth 195:261–269. 10.1016/j.jneumeth.2010.11.015 PMC3244474 21130115

[14] Kamiński M, Blinowska KJ (1991) A new method of the description of the information flow in the brain structures. Biol Cybern 65:203–210. 1912013 10.1007/BF00198091

[15] Franaszczuk PJ, Blinowska KJ, Kowalczyk M (1985) The application of parametric multichannel spectral estimates in the study of electrical brain activity. Biol Cybern 51:239–247. 3970984 10.1007/BF00337149

[16] Ding M, Bressler SL, Yang W, Liang H (2000) Short-window spectral analysis of cortical event-related potentials by adaptive multivariate autoregressive modeling: Data preprocessing, model validation, and variability assessment. Biol Cybern 83:35–45. 10933236 10.1007/s004229900137

[17] Blinowska KJ (2008) Methods for localization of time-frequency specific activity and estimation of information transfer in brain. International Journal of Bioelectromagnetism 10:2–16.

[18] Chen D, Li H, Yang Y, Chen J (2012) Causal Connectivity Brain Network: A novel method of motor imagery classification for brain–computer interface applications. 2012 International Conference on Computing, Measurement. Control and Sensor Network 23:87–90.

[19] Yuan H, Liu T, Szarkowski R, Rios C, Ashe J, et al (2010) Negative covariation between task-related responses in alpha/beta-band activity and BOLD in human sensorimotor cortex: An EEG and fMRI study of motor imagery and movements. NeuroImage 49:2596–2606. 19850134 10.1016/j.neuroimage.2009.10.028 PMC2818527

[20] Ball T, Schreiber A, Feige B, Wagner M, Lücking CH, et al (1999) The role of higher-order motor areas in voluntary movement as revealed by high-resolution EEG and fMRI. NeuroImage 10:682–694. 10600414 10.1006/nimg.1999.0507

[21] Seth AK (2010) A MATLAB toolbox for Granger causal connectivity analysis. J Neurosci Meth 186:262–273. 10.1016/j.jneumeth.2009.11.020 19961876

[22] Buchsbaum MS (2004) Frontal Cortex Function. Am J Psychiatry 161:2178–2178. 15569885 10.1176/appi.ajp.161.12.2178

[23] Hétu S, Grégoire M, Saimpont A, Coll MP, Eugène FP, et al (2013) The neural network of motor imagery: an ALE meta-analysis. Neurosci Biobehav Rev 37:930–949. 23583615 10.1016/j.neubiorev.2013.03.017

[24] Pfurtscheller G, Lopez-da-Silva FH (1999) Event-related EEG/MEG synchronization and esynchronization: basic principles. Clin Neurophysiol 110:1842–1857. 10576479 10.1016/s1388-2457(99)00141-8

[25] Klimesch W (1996) Memory processes, brain oscillations and EEG synchronization. Int J Psychophysiol 24:61–100. 8978436 10.1016/s0167-8760(96)00057-8

[26] Klimesch W, Doppelmayr M, Pachinger T, Russegger H (1997) Event-related desynchronization in the alpha band and the processing of semantic information. Cogn Brain Res 6:83–94. 10.1016/s0926-6410(97)00018-9 9450602

[27] Klimesch W, Doppelmayr M, Russegger H, Pachinger T (1996) Theta band power in the human scalp EEG and the encoding of new information. NeuroReport 7:1235–1240. 8817539 10.1097/00001756-199605170-00002

[28] Klimesch W (1999) EEG alpha and theta oscillations reflect cognitive and memoryperformance: a review and analysis. Brain Res Rev 29:169–195. 10209231 10.1016/s0165-0173(98)00056-3

[29] Bedny M, Caramazza A, Grossman E, Pascual-Leone A, Saxe R (2008) Concepts are more than percepts the case of action verbs. J Neurosci 28:11347–11353. 18971476 10.1523/JNEUROSCI.3039-08.2008 PMC2752357

