# Supplementary figures and images for: Evaluation of EEG Oscillatory Patterns and Cognitive Process during Simple and Compound Limb Motor Imagery
Source: PLoS One. 2014 Dec 9;9(12):e114853. doi: 10.1371/journal.pone.0114853 (PMC4260914; doi:10.1371/journal.pone.0114853)

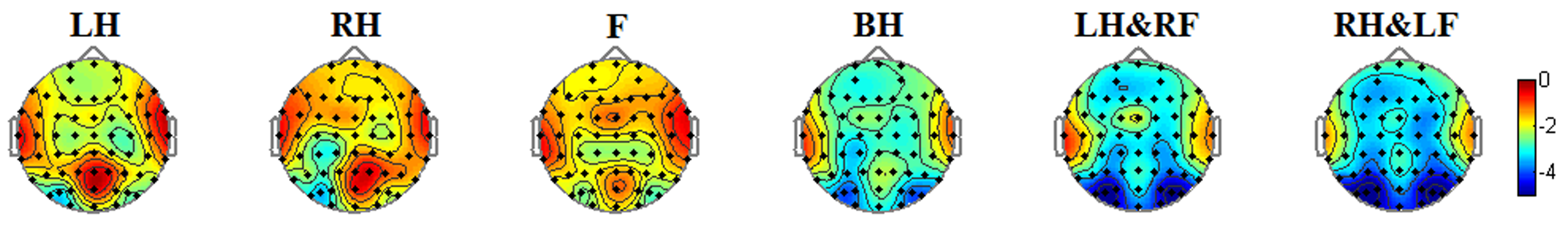

Supplement: S1 Figure — The topographical distribution of 10–11Hz ERD for six MI tasks. LH, RH, F, BH, LH&RF, RH&LF indicate left hand, right hand, feet, both hands, left hand combined with right foot, right hand combined with left foot respectively. Blue regions indicate the involved areas when ERD occurs during mental tasks. (TIF) [file pone.0114853.s001.tif]

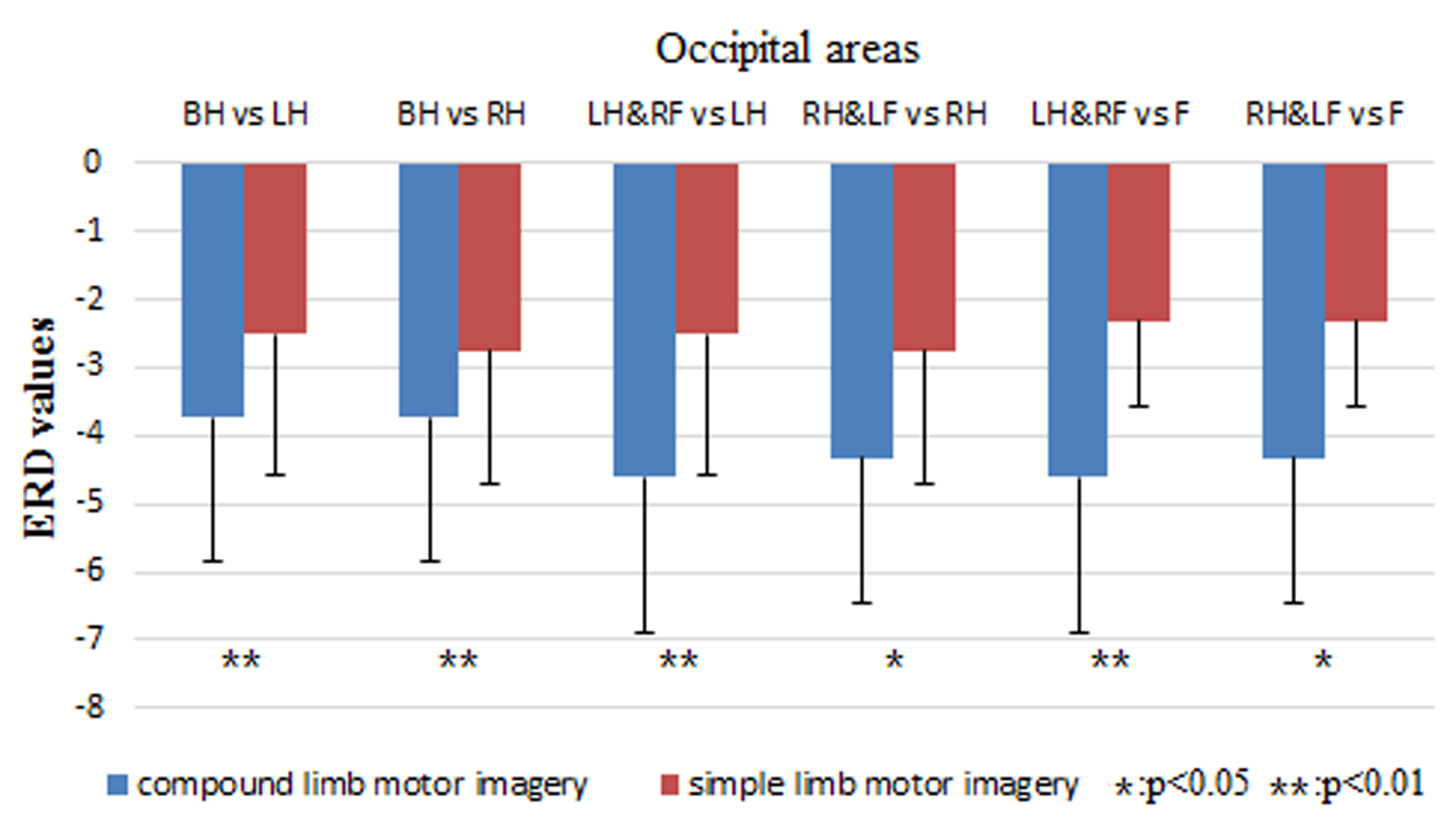

Supplement: S2 Figure — The comparison of ERD values in six groups for occipital areas. Blue bar indicates compound limb motor imagery, while red bar indicates simple limb motor imagery. Condition pairs that significantly differ from each other are indicated by an asterisk (p<0.05) or two asterisks (p<0.01). (TIF) [file pone.0114853.s002.tif]

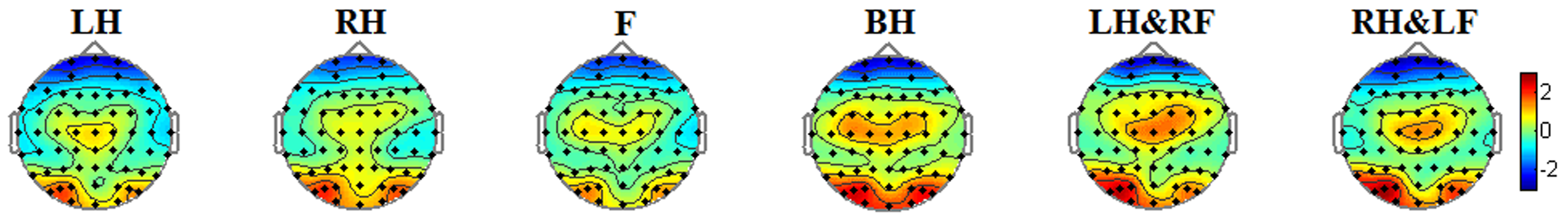

Supplement: S3 Figure — The topographical distribution of ERS patterns within theta band for six MI tasks. LH, RH, F, BH, LH&RF, RH&LF indicate left hand, right hand, feet, both hands, left hand combined with right foot, right hand combined with left foot respectively. Red regions indicate the involved areas when ERS occurs during mental tasks. (TIF) [file pone.0114853.s003.tif]

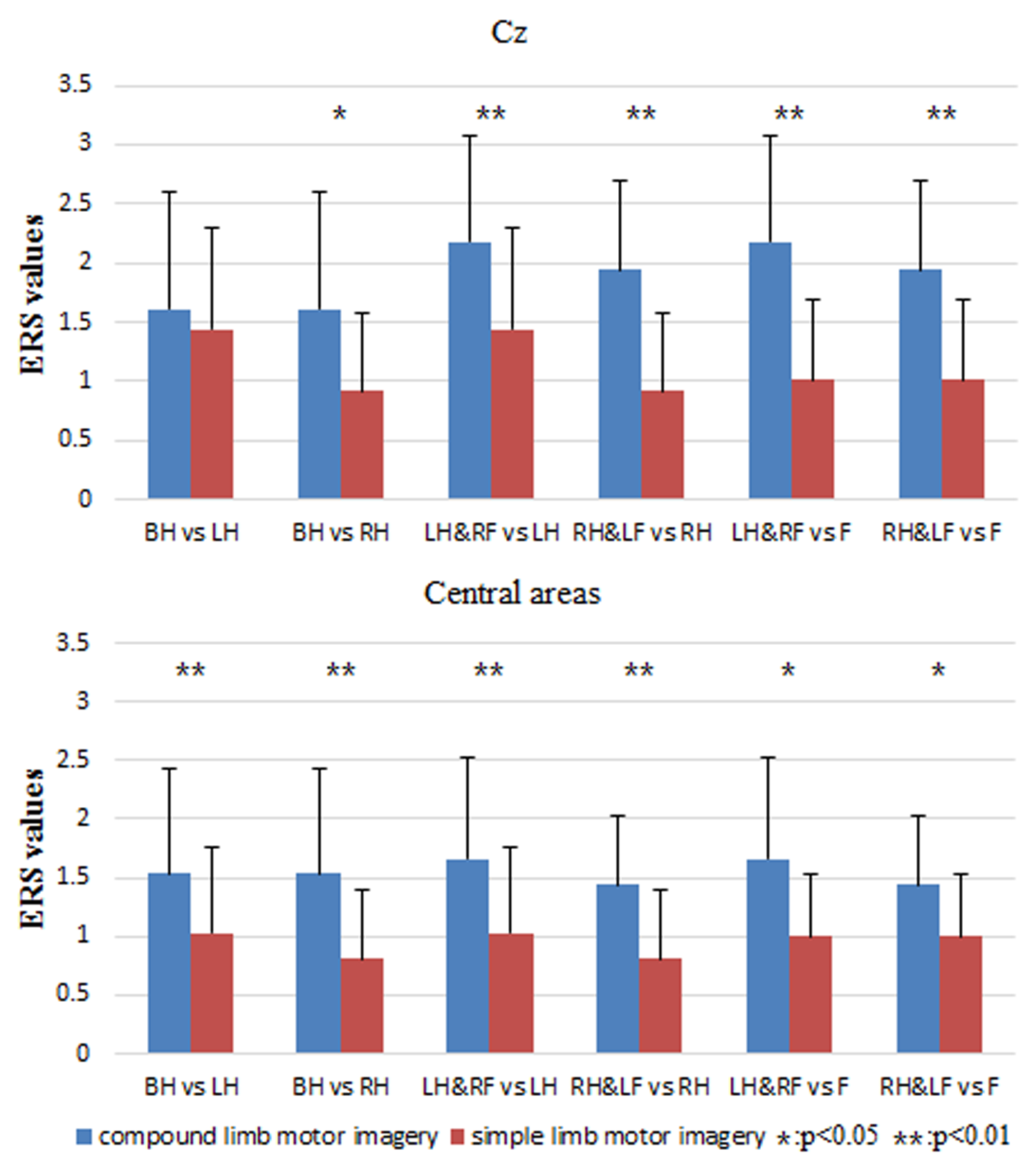

Supplement: S4 Figure — The comparison of ERS values in six groups for Cz and central areas. Blue bar indicates compound limb motor imagery, while red bar indicates simple limb motor imagery. Condition pairs that significantly differ from each other are indicated by an asterisk (p<0.05) or two asterisks (p<0.01). (TIF) [file pone.0114853.s004.tif]

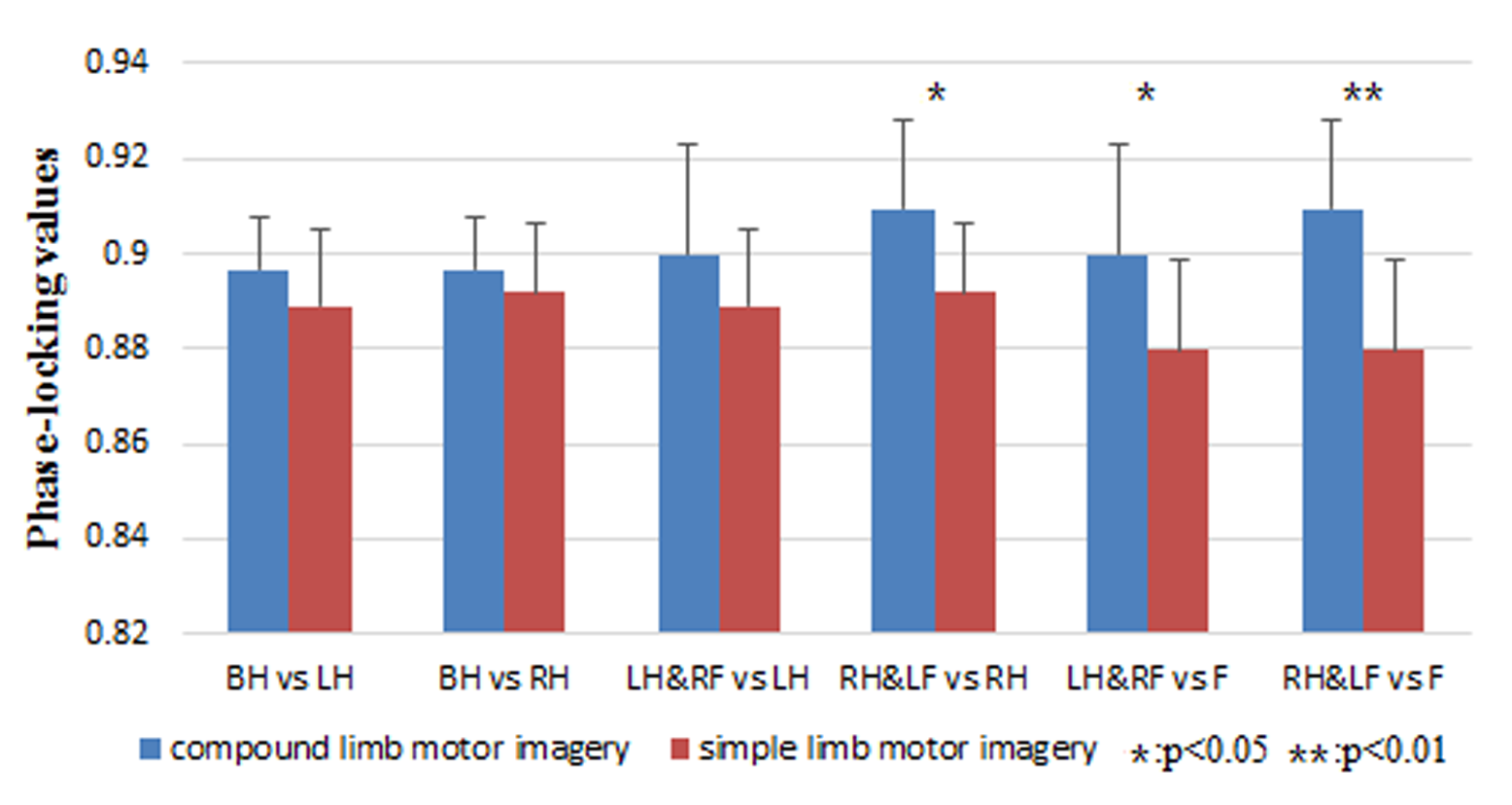

Supplement: S5 Figure — The comparison of the PLV between central and occipital areas in six groups. Blue bar indicates compound limb motor imagery, while red bar indicates simple limb motor imagery. Condition pairs that significantly differ from each other are indicated by an asterisk (p<0.05) or two asterisks (p<0.01). (TIF) [file pone.0114853.s005.tif]
